# Calendar

**DOI:** 10.1155/S1463924689000295

**Published:** 1989


					j. A. Ibafiez et al. Automation of ion-selective electrode measurements
this time, the associated electrical potential difference
and, finally, the temperature.
Conclusion
The technique described in this paper is an efficient and
reliable method for measuring concentration changes
with ISEs. The incorporation of a sample changer with
measuring cell, thermostated in parallel with the reactor,
allows the periodic calibration of ISEs and the measure-
ment ofproblem concentrations at the same temperature,
and can therefore eliminate errors.
Acknowledgement
The authors acknowledge financial support from the
CICYT of the Spanish government under grant No.
PB85-0240-C02-02.
References
1. IBAv.z,J. A. VICTORIA, L., ORTEGA-NAvAS,J. HERNNDEZ,
A. and TEJERINA, F., Anales de Ffsica (B), 82 (1986), 213.
Calendar
JUNE
25-30 June 1989 Eurosensors III and 5th International
Conference on Sensors and Actuators: Montreux, Switzerland.
Contact Eurosensors (Transducers '89), COMST S.A.,
PO Box 415, 1001 Lausanne 1, Switzerland.
JULY
30 July-5 August 1989 SAC 89: Cambridge, UK. Contact
Analytical Division, Royal Society ofChemistry, Burling-
ton House, Piccadilly, London W1V 0BN.
SEPTEMBER
11-13 September 1989 RSC/SCI/IOP Surface Analysis
Techniques and Applications: Manchester, UK. Contact Mrs
E. S. Wellingham, Field End House, Bude Close, Nailsea,
Bristol BS 19 2FQ.
20-22 September 1989 International Symposium on Detection
in Liquid Chromatography and Flow Injection Analysis: Cdr-
doba, Spain. Contact Dr M. D. Luque de Castro, Dpto.
Quimica Analitica, Facultad de Ciencias, 14004 C6r-
doba, Spain.
25-27 September 1989 Sensors and their Applications IV:
Canterbury, UK. The Meetings Officer, Institute of
Physics, 47 Belgrave Square, London SW1X 8QX.
25-28 September 1989 Third International Symposium on
Kinetics in Analytical Chemistry: Dubrovnik, Yugoslavia. Con-
tact Professor Gordana A. Milonovi(:. Department of
Chemistry, University of Belgrade, KAC PO Box 550,
11001 Belgrade, Yugoslavia.
26-28 September 1989 The British Laboratory Week:
Olympia, London. Including Laboratory 89, Computer
Aided Sciences, Bio 89, Medical Laboratory Sciences and
Analyticon. Contact Curtis Steadman and Partners, The
Hub, Emson Close, Saffron Walden, Essex CB 10 1HL.
1990
MARCH
5-9 March 1990 41st Pittsburgh Conference and Exposition on
Analytical Chemistry and Applied Spectroscopy: New York,
USA. Contact The Pittsburgh Conference, 300 Penn
Center Boulevard, Suite 332, Pittsburgh PA 15235, USA.
AUGUST
26-31 August 1990 Euroanalysis VII: Vienna, Austria.
Contact Prof. Dr M. Grasserbauer, c/o Interconvention,
Austria Center Vienna, A-1450 Vienna, Austria.
133

				

